# The GMOseek matrix: a decision support tool for optimizing the detection of genetically modified plants

**DOI:** 10.1186/1471-2105-14-256

**Published:** 2013-08-22

**Authors:** Annette Block, Frédéric Debode, Lutz Grohmann, Julie Hulin, Isabel Taverniers, Linda Kluga, Elodie Barbau-Piednoir, Sylvia Broeders, Ingrid Huber, Marc Van den Bulcke, Petra Heinze, Gilbert Berben, Ulrich Busch, Nancy Roosens, Eric Janssen, Jana Žel, Kristina Gruden, Dany Morisset

**Affiliations:** 1 Bavarian Health and Food Safety Authority (LGL), Oberschleißheim, Germany; 2 Walloon Agricultural Research Centre (CRA-W), Gembloux, Belgium; 3 Federal Office of Consumer Protection and Food Safety (BVL), Berlin, Germany; 4 Institute for Agricultural and Fisheries Research (EV ILVO), Merelbeke, Belgium; 5 European Commission, Directorate General Joint Research Centre, Institute for Health and Consumer Protection (EC JRC-IHCP), Ispra, Italy; 6 Scientific Institute of Public Health (WIV-ISP), Brussels, Belgium; 7 National Institute of Biology (NIB), Ljubljana, Slovenia

**Keywords:** Genetically modified organism (GMO), GMO screening, Matrix approach, Genetically modified plant

## Abstract

**Background:**

Since their first commercialization, the diversity of taxa and the genetic composition of transgene sequences in genetically modified plants (GMOs) are constantly increasing. To date, the detection of GMOs and derived products is commonly performed by PCR-based methods targeting specific DNA sequences introduced into the host genome. Information available regarding the GMOs’ molecular characterization is dispersed and not appropriately organized. For this reason, GMO testing is very challenging and requires more complex screening strategies and decision making schemes, demanding in return the use of efficient bioinformatics tools relying on reliable information.

**Description:**

The GMOseek matrix was built as a comprehensive, online open-access tabulated database which provides a reliable, comprehensive and user-friendly overview of 328 GMO events and 247 different genetic elements (status: 18/07/2013). The GMOseek matrix is aiming to facilitate GMO detection from plant origin at different phases of the analysis. It assists in selecting the targets for a screening analysis, interpreting the screening results, checking the occurrence of a screening element in a group of selected GMOs, identifying gaps in the available pool of GMO detection methods, and designing a decision tree. The GMOseek matrix is an independent database with effective functionalities in a format facilitating transferability to other platforms. Data were collected from all available sources and experimentally tested where detection methods and certified reference materials (CRMs) were available.

**Conclusions:**

The GMOseek matrix is currently a unique and very valuable tool with reliable information on GMOs from plant origin and their present genetic elements that enables further development of appropriate strategies for GMO detection. It is flexible enough to be further updated with new information and integrated in different applications and platforms.

## Background

Genetically modified organisms (GMOs) of plant origin have been widely approved for commercialisation at a global scale [1,2]. To allow consumer freedom of choice, many countries regulate the authorization, labelling, and compliance control of GMOs. The constantly increasing number of GMOs on the world market raises a new major challenge for testing laboratories: limiting the cost increase while meeting all test criteria and requirements demands higher throughput to keep GMO monitoring efficient and cost-affordable for enforcement purposes.

To date, PCR-based detection methods are the standards to routinely analyse the GMO presence in the food/feed chain [3-6]. Approaches for targeting DNA sequences of GMOs in food and feed samples can be based on the detection of commonly used genetic elements (or groups of genetic elements) for screening purposes (screening tests) [4] or solely on the identification of the specific GMO events (event-specific tests) [7]. For the screening approach, the choice of tests covering a wide range of GMOs has become less obvious and is hampered by the lack of available information regarding the genetic elements introduced into the different GMOs. The use of event-specific tests is extremely costly given the high number of GMO events to be scrutinized, and it only enables the detection of known events.

The common strategy followed by laboratories in the European Network of GMO Laboratories (ENGL) is based on the so-called “matrix approach” [8,9] which combines both types of tests [10-13]. A first step consists of screening tests chosen to allow large coverage of GMOs. By comparing the test results with tabulated data describing the presence/absence of the targeted sequences in individual events (matrix), the analyst evaluates the possible presence of individual GMO events. Confirmation and identification of GMOs in a second step is performed using event-specific tests. When a screening test pattern cannot be explained by a registered GMO, a sample is considered as non-compliant and additional testing (e.g. insertional cloning or genome walking combined with DNA sequence analysis) is required to elucidate the origin of the unexplained positive signals [5,9,13,14].

A matrix-based screening approach enables the smart selection of a minimum set of tests that can cover a maximum number of GMOs, thus reducing the number of costly event-specific tests to be performed. A major requisite for applying a “matrix approach” in GMO detection is the availability of reliable information on the molecular targets present in the GMOs under investigation [10]. Existing databases mainly collect the information for GMO risk assessment [15-18] and the methods available for detection [19,20]. Several target/GMO tables demonstrate the use of the matrix approach in GMO analysis [21-25]. In most cases, the scope of the GMOs and the listed targets have been restricted, such as for the GMOs authorized for commercial use in the European Union (EU) at a particular time. Moreover, most of the existing datasets are not presented in a format appropriate for decision-making in GMO detection, or data are missing because some early attempts utilized a limited amount of data.

The main purpose of the present work was to fill these essential gaps by building an extensive database on GMOs and their genetic elements (hereafter named “GMO elements”) by the partners of the GMOseek project. The aim of the GMOseek matrix is to focus on the genetically modified plants that can be found in food or feed products. The information compiled in the GMOseek matrix originates from all available sources at the time of building it. When the detection method and (certified) reference material were available, data were also experimentally verified. The GMOseek matrix is presented with effective functionalities for flexible data retrieval in a format enabling easy modification and addition of new information and even implementation into larger database platforms. The GMOseek matrix facilitates a quick, easy overview of all genetic elements and their presence or absence in numerous GMO events that can be found on the global market.

### Construction and content

#### Data collection

Data on GMO events and GMO elements collected in the GMOseek matrix originate from various sources, primarily the Centre for Biosafety and Sustainability (BATS) report [26] and the Centre for Environmental Risk Assessment (CERA) database [16]. Detailed information on authorized GMO events or GMOs undergoing EU commercialization was obtained from scientific opinions on applications of the European Food Safety Authority (EFSA) [27], safety assessment reports, and EU market authorization notifications for GM products in accordance with regulation 1829/2003/EC and the directive 2001/18/EC [28]. For quality control, these publicly available data were verified on a documentary basis by the Institute for Health and Consumer Protection, Joint Research Centre (JRC-IHCP), which is the EU Reference Laboratory for Genetically Modified Food and Feed (EU-RL GMFF) and a partner in the GMOseek project. The approval status of GMO events was obtained from the DG Health and Consumers website [28]. Relevant data were also collected from publicly available documents provided by public authorities in other jurisdictions: market authorization applications for GM products in New Zealand/Australia [29], consultations on bioengineered foods at the U.S. Food and Drug Administration [30], petitions for de-regulation at the U.S. Department of Agriculture [31], and the Japan Biosafety Clearing House [32]. Online databases such as the GMO-Compass database [17] and the GMO detection database [19] also provided important information regarding GMO genetic composition. A table developed by the team of Changming Lu [20] listing 101 GMOs and some elements present in their construction was also consulted. Several patent search engines [33-35] and nucleotide sequence databases of the National Center for Biotechnology Information [36] were used to obtain detailed information on the transgenic sequences. Published research studies [37-41] were useful for GM events without any available official documentation. Some publications [13,42,43] provided informative summary tables on experimental verifications of screening targets.

The GMO plant events listed in the matrix include those currently or previously authorized in the EU, those that are in the pipeline for commercialization worldwide, and GMO events that were previously accidentally found on the market. GMOs designed for pure research purposes are not listed in the matrix. For GM rice, events involved in a preproduction step in China are also listed because they may be found worldwide, as is true for several other GM rice events [44-47].

#### Data assembly and quality control

For quality control, all collected data were tabulated in an Excel environment (Additional file 1, GMOseek_Matrix-Program_version_13-1) and independently verified by at least two partners of the project. In cases of conflicting information on data from different sources, the priority was given to official documents provided by the company and experimental verification (if possible or referenced) to solve the ambiguity. Most conflicting data were explained by differences in nomenclature or because genetic elements present in the vector used for transformation were excluded from or only partially integrated into the plant genome. As similar names can be used for GM elements originating from different species, the terminology and abbreviations were differentiated accordingly. For example, the promoters pActin1 from rice (present in NK603 maize), pActin2 from *Arabidopsis thaliana* (present in COT102 cotton), and pActin2 from rice (described in US patent 6,429,357) were distinguished. To avoid errors and misunderstanding, only one terminology was used to describe the same genetic element (e.g. p35S includes pE35S, and pFMV includes pFMV34S, pCmoVb, peFMV), or the same event (e.g. rapeseed with unique identifier ACS-BN007-1 is known as HCN92 and Topas 19/2).

**Figure 1:**
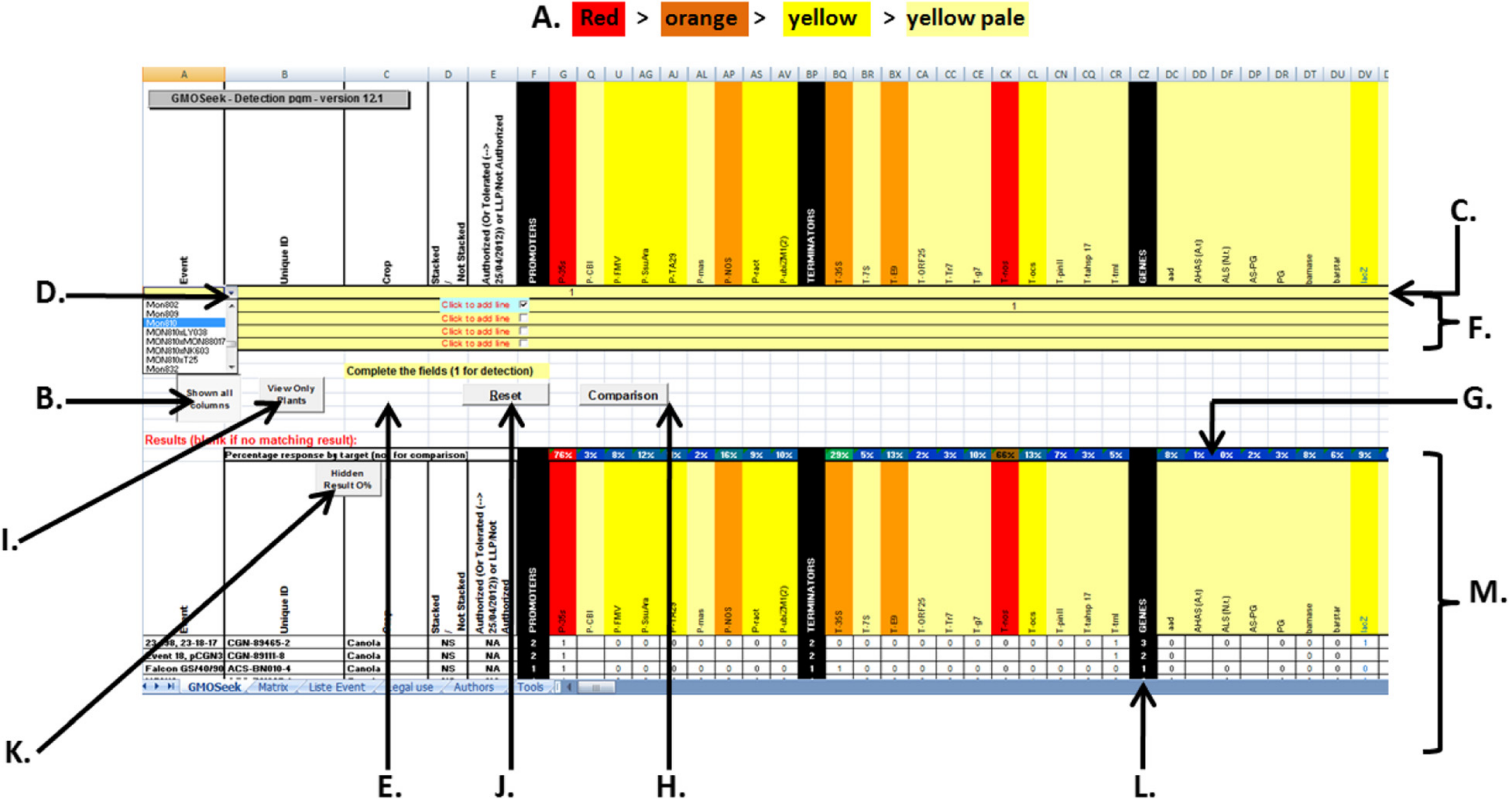
**An overview of the display and functionalities of the GMOseek excel application.** The GMOseek matrix contains is a tabulated database with associated macros that permit an easy search of information. The Figure 1 present an overview of some functionalities present in the GMOseek matrix. **A.** Color code indicating the frequency of occurrence of elements in events in the whole database (red ≥30%, orange between 29 and 10%, yellow between 9% and 5%, pale yellow between 4% and 2%, uncoloured cells ≤ 2%), **B.** Click on the button to see all screening elements or only the most representative ones, **C.** Row where data on presence or absence (1 or 0) per parameter can be indicated, **D.** Drop down menu in the first five columns, **E.** Location of the sorting button (no longer visible once the search are displayed), **F.** Extra rows to activate if several profiles are introduced, **G.** Frequency of occurrence of the screening elements in the results of the selection, **H.** For comparison, enter new parameters and click on comparison, **I.** Direct access to the data concerning plant targets, **J.** Reset before a new search, **K.** Hide results with 0% frequency, **L.** Black columns separate sections (e.g. promoters, terminators,…) and give the amount of elements within this section, **M.** Results of the search. A complete description of the functionalities is present in the user manual.

The GMOseek matrix is an Excel-based database with different sets of information grouped into different worksheets. Information on all GMO events and data that have been compiled during the GMOseek project is stored in a worksheet entitled “Matrix”. In this sheet, general information is provided regarding the trade name, unique identifier [48], crop species, type of event (single or stacked), and the EU authorization status (authorized, not authorized, or falling under the Commission regulation (EU) No 619/2011 [49]). The status of authorization specified in the GMOseek matrix is the one available for the EU at the release date of the latest matrix version (18/07/2013) and can be adapted by the user according to the relevant jurisdiction. In the same worksheet, information on the presence or absence of the different GMO elements is provided, grouped in five sections consisting of 55 promoters, 31 terminators, 103 open-reading frame segments, 47 miscellaneous elements (e.g. vector elements, enhancers), and 11 junctions between elements in the transgene constructs. A sixth section contains information describing 28 endogenous plant targets. The presence and absence of a given GMO element or an endogenous target is marked by 1 and 0, respectively. For those events for which very limited data were available, respective cells concerning the elements were left empty. These cells will be filled in updated versions if complementary information becomes available. A colour code of the column headers is used for visual indication of the frequency of the GMO element occurrence in all listed GMO events (Figure 1, item A).

#### Experimental verification of data

As an additional step of quality control, data in the GMOseek matrix were experimentally verified when relevant screening detection methods and (certified) reference materials were available. The presence/absence of GMO elements was experimentally tested by quantitative real-time PCR (qPCR) using CRMs mainly available from the EC-JRC Institute for Reference Materials and Measurements (IRMM, Geel, Belgium) or the American Oil Chemists’ Society (AOCS, Urbana, IL, USA). Individual reference materials are listed in the Table 1.

**Table 1:** **QPCR results obtained with screening targets** (**TaqMan probes**) **on AOCS and IRMM certified reference material**

**A. Screening targets (promoters and terminators)**																		
**Crop species**	**GM events**	**Reference material**	**p35S**[50]	**p35S**[51]	**p35S**[52]	**pFMV**[53]	**pNOS**[53]	**pUbi**[53]	**pSsuAra**[53]	**pTA29**[53]	**pRice Actin**[53]	**tE9**[53]	**tNOS**[52]	**tNOS**[39]	**t35S**[53]	**tOCS**[53]	**tg7**[53]	**Source of impurity**
**Rapeseed**	GT73 (>99%)	AOCS 0304-B	-	-	-	+	-	N	-	-	N	+	-	-	-	-	-	/
	T45 (100%)	AOCS 0208-B	+	+	+	-	-	N	-	-	N	-	-	U^(+)^	U^(+)^	-	-	not identified
	MS8 (100%)	AOCS 0306-F2	-	-	-	-	-	N	+	+	N	-	+	+	-	-	+	/
	RF3 (100%)	AOCS 0306-G	-	-	-	-	-	N	+	+	N	-	+	+	-	-	+	/
	Topas 19/2 (100%)	Bayer REF-0019/04	N	+	+	N	N	N	N	N	N	N	U^(+)^	-	N	N	N	not identified
**Soybean**	GTS 40-3-2 (10%)	ERM-BF410gk	+	+	+	-	N	N	N	N	N	-	+	+	-	N	N	/
	MON89788 (>99%)	AOCS 0906-B	R^+^	R^+^	-	+	N	N	N	N	N	+	R^+^	-	-	N	N	GTS 40-3-2
	A2704-12 (100%)	AOCS 0707-B	+	+	+	-	N	N	N	N	N	-	-	-	+	N	N	/
	A5547-127 (100%)	AOCS 0707-C2	+	+	+	-	N	N	N	N	N	-	-	-	+	N	N	/
	305423 (10%)	ERM-BF426d	R^+^	R^+^	R^+^	-	N	N	N	N	N	-	R^+^	R^+^	-	N	N	GTS 40-3-2
	356043 (1%)	ERM-BF425c	R^+^	-	R^+^	-	N	N	N	N	N	-	R^+^	-	-	N	N	GTS 40-3-2
**Maize**	GA21 (4.29%)	ERM-BF414f	R^+^	-	-	-	N	N	N	N	+	N	+	+	-	N	N	Bt176/ MON810
	MON810 (5%)	ERM-BF413f	+	+	+	-	N	N	N	N	-	N	-	-	-	N	N	/
	Bt176 (1%)	ERM-BF411d	+	+	+	-	N	N	N	N	-	N	-	-	BS	N	N	/
	TC 1507 (9.85%)	ERM-BF418d	+	+	+	-	N	N	N	N	-	N	-	-	+	N	N	/
	T25 (100%)	AOCS0306-H	+	+	+	-	N	N	N	N	-	N	-	-	+	N	N	/
	NK603 (1.96%)	ERM-BF415e	+	+	+	-	N	N	N	N	+	N	+	+	-	N	N	/
	DAS59122 (0.1%)	ERM-BF424b	+	+	+	-	N	N	N	N	-	N	-	-	U^(+)^	N	N	/
	MON88017 (100%)	AOCS 0406-D	+	+	+	-	N	N	N	N	+	N	+	+	-	N	N	/
	MON89034 (98%)	AOCS 0906-E	+	+	+	+	N	N	N	N	R^+^	N	+	+	-	N	N	MON88017
	CBH351 (1%)	Sigma-A. 69407	+	+	+	-	N	N	N	N	-	N	+	+	U^(+)^	N	N	/
	3272 (9.8%)	ERM-BF420c	-	-	-	-	N	N	N	N	-	N	+	+	U^-^	N	N	/
	98140 (10%)	AOCS 0607-A	U^-^	U^-^	+	-	N	N	N	N	-	N	-	-	-	N	N	/
	MIR604 (9.85%)	ERM-BF423d	-	-	-	-	N	N	N	N	-	N	+	+	-	N	N	/
	MON863 (9.85%)	ERM-BF416d	+	+	+	-	N	N	N	N	-	N	+	+	-	N	N	MON810 (Table 1.B)
**Cotton**	MON1445 (100%)	AOCS 0804-B	+	+	+	+	N	-	N	N	N	+	+	+	N	N	N	/
	LL25 (100%)	AOCS 0306-E	+	N	N	-	N	-	N	N	N	-	+	N	N	N	N	/
	GHB119 (10%)	ERM-BF428c	+	N	N	-	N	-	N	N	N	-	+	N	N	N	N	/
	281x3006 (>97%)	ERM-BF422b	-	-	-	R^+^	N	+	N	N	N	R^+^	-	-	N	N	N	MON1445
	MON531 (100%)	AOCS 0804-C	+	+	+	R^+^	N	-	N	N	N	R^+^	+	+	N	N	N	MON1445
	MON15985 (100%)	AOCS 0804-D	+	+	+	R^+^	N	-	N	N	N	R^+^	+	+	N	N	N	MON1445
**B. Screening targets (genes and junctions)**																		
**Crop species**	**GM events**	**Reference material**	**EPSPS1**	**EPSPS2**[54]	**gox**	**bar**	**bar**[54]	**pat**[54]	**p35S-bar**[55]	**p35S-hsp70**[56]	**p35S-pat**[51]	***CTP2-*cp4epsps**[57]	***cry*1Ab**	**Source of impurity**				
**Rapeseed**	GT73 (>99%)	AOCS 0304-B	-	+	+	-	-	-	-	-	-	+	N	/				
	T45 (100%)	AOCS 0208-B	-	-	-	-	-	+	(+)	U^-^	+	0	N	not identified				
	MS8 (100%)	AOCS 0306-F2	-	-	-	+	+	-	+	-	-	-	N	/				
	RF3 (100%)	AOCS 0306-G	-	-	-	+	+	-	-	-	-	-	N	/				
	Topas 19/2 (100%)	Bayer REF-0019/04	N	N	N	N	-	+	-	-	+	-	N	not identified (Table 1.A)				
**Soybean**	GTS 40-3-2 (10%)	ERM-BF410gk	+	-	N	-	-	-	-	-	-	-	N	/				
	MON89788 (>99%)	AOCS 0906-B	R^+^	+	N	-	-	-	-	-	-	+	N	GTS 40-3-2				
	A2704-12 (100%)	AOCS 0707-B	-	-	N	-	-	+	-	-	+	-	N	/				
	A5547-127 (100%)	AOCS 0707-C2	-	-	N	-	-	+	-	-	+	-	N	/				
	305423 (10%)	ERM-BF426d	R^+^	-	N	-	-	-	N	N	-	-	N	GTS 40-3-2				
	356043 (1%)	ERM-BF425c	R^+^	-	N	-	-	-	N	N	-	-	N	GTS 40-3-2				
**Maize**	GA21 (4.29%)	ERM-BF414f	-	-	N	R^+^	-	-	R^-^	+	-	-	+	Bt176/ MON810				
	MON810 (5%)	ERM-BF413f	-	-	N	-	-	-	-	+	-	-	+	/				
	Bt176 (1%)	ERM-BF411d	-	-	N	+	+	-	+	-	-	-	+	/				
	TC 1507 (9.85%)	ERM-BF418d	-	-	N	-	-	+	-	-	+	-	-	/				
	T25 (100%)	AOCS 0306-H	-	-	N	-	-	+	-	-	+	-	-	/				
	NK603 (1.96%)	ERM-BF415e	+	-	N	-	-	-	-	+	-	+	-	/				
	DAS59122 (0.1%)	ERM-BF424b	-	-	N	-	-	+	-	-	+	-	-	/				
	MON88017 (100%)	AOCS 0406-D	+	-	N	-	-	-	-	-	-	+	-	/				
	MON89034 (98%)	AOCS 0906-E	-	R^+^	N	-	-	-	-	-	-	-	+	MON88017				
	CBH351 (1%)	Sigma-A. 69407	-	-	N	+	+	-	+	-	-	-	-	/				
	3272 (9.8%)	ERM-BF420c	-	-	N	-	-	-	-	-	-	-	-	/				
	98140 (10%)	AOCS 0607-A	-	-	N	-	-	-	N	N	-	-	-	/				
	MIR604 (9.85%)	ERM-BF423d	-	-	N	-	-	-	-	-	-	-	-	/				
	MON863 (9.85%)	ERM-BF416d	-	-	N	-	-	-	-	R^+^	-	-	-	MON810				
**Cotton**	MON1445 (100%)	AOCS 0804-B	-	+	N	-	-	-	N	N	-	+	N	/				
	LL25 (100%)	AOCS 0306-E	-	-	N	+	N	N	N	N	N	N	N	/				
	GHB119 (10%)	ERM-BF428c	-	-	N	+	N	N	N	N	N	N	N	/				
	281x3006 (>97%)	ERM-BF422b	-	R^+^	N	-	-	+	-	-	-	+	N	MON1445				
	MON531 (100%)	AOCS 0804-C	-	R^+^	N	-	-	-	N	N	-	+	N	MON1445				
	MON15985 (100%)	AOCS 0804-D	-	R^+^	N	-	-	-	N	N	-	+	N	MON1445				

GM events: Name of the GM events and its mass/mass content into the tested reference material (in brackets). Reference material: Catalogue reference of the reference material. Sigma-A. = Sigma-Aldrich. Positive and negative screening results are indicated by + and -, respectively. Late signals, with Cq > 37 are indicated by the plus sign between brackets, (+). Unexpected positive and negative results are indicated by U^+^ and U^-^, respectively; false positive results due to GM impurities in the reference material (confirmed by event-specific tests) are indicated by R^+^. BS = bad signal and amplification curves; N = not tested. Late signals and bad amplification curves are presumed to be due to differences of sequences affecting the efficiency of the PCR and the hybridization of the primers and probes. The positive signal for MON89034 maize with *cry*1Ab primers and probe, indicated by a bold +, is due to the presence of *cry*1A105 having a sequence similar to the targeted region.

DNA was extracted with the CTAB method following the recommendations of the ISO21571 standard [58] and tested with qPCR using TaqMan probes. Targets used for this verification test included pUbi, pNOS, tE9, pFMV, pRice pActin, pSsuAra, pTA29, t35S, tOCS, tg7 [53], *cry*1Ab, bar, EPSPS-1, gox (Debode, unpublished), EPSPS-2 [54], CTP2-cp4epsps [57], pat [54], the junction p35S-bar [55], the junction p35S-pat [56], the junction p35S-hsp70 [51], an additional bar sequence [57], three different sequences of p35S [50,52,59], and two sequences of tNOS [39,52]. Contaminations were checked with the available ring-trial validated event-specific methods [60].

### Utility and discussion

The GMOseek database tabulates the presence or absence of 247 genetic elements within an array of 328 GMO events. Typical GMO elements include regulatory sequences (e.g. promoters, terminators), coding sequences of reporter or selective resistance genes (e.g. antibiotic resistance genes), and genes responsible for new traits (herbicide tolerance, insect resistance, special metabolic properties). Some common junctions between frequently used elements are also listed, because these junctions are considered as potential screening targets and may be suitable for the development of construct-specific screening tests.

So far, the information contained in public databases is either limited in terms of the listed GMO events, either presented in a format not easily exportable for update, modification and/or customization. The GMOseek database is an Excel application to enable accessible information and the associated functionalities for the largest interested audience. It can be easily modified and exported for update or customized use. All data available were collected and carefully evaluated. Additionally, data were experimentally verified when CRMs and detection methods were available, resulting in a comprehensive and reliable database. Besides the matrix itself, this database provides effective functionalities supporting different steps in routine GMO analysis as well as in the development of new detection strategies.

#### Functionalities of the GMOseek matrix

Applications enabling functionalities are located on a spreadsheet independent from the ‘Matrix‘ sheet of the GMOseek database (Figure 1). Detailed instructions on the use of these functionalities are given in the user manual (Additional file 2, User Manual GMOseek Matrix).

To allow a fast and easy search for information within the matrix, a set of four colours was introduced to provide visual information on the frequency of GMO elements within the listed GMO events (Figure 1, item A). The user is also offered the opportunity to interrogate and sort the database (Figure 1, item B) according to several parameters that can be combined in the same request. To do so, a series of drop-down menus displays a list of selectable characteristics classified in alphabetical order (Figure 1, item C). This allows the user to select an appropriate GMO event, Unique ID, crop, stacked or non-stacked GM, and status of authorization.

A third type of search can be performed by indicating the presence or absence of one or several GMO elements as request parameter (Figure 1, item D). Events with a defined GMO element composition are searched by filling in either 1 (mandatory presence of GMO element in the retrieved GMO events) or 0 (exclude GMO events having that GMO element), thereby generating a subset of fitted GMO events. If several values for several GMO elements are introduced as a request in the same line, the “AND” function is utilized (e.g. all the GMO events having both p35S and tNOS). For retrieval of the GMO events having at least one of the selected GMO elements (e.g. GMO events with p35S or tNOS), appropriate alternative parameters can be introduced in a separate line utilizing thereby the “OR” function (Figure 1, item E). For more convenience, this type of search by presence/absence of GMO elements can also be combined with a selection of defined parameters allowed by the top-down menus.

Another functionality allows the comparison of two GMO events, or of a single GMO event and a combination of genetic elements (Figure 1, item F). This functionality allows the operator to visually check the similarities or differences between two GMOs or between a GMO and a sorting result, such as a screening assay.

Independently of its type (top-down menus, absence/presence of GMO elements, comparison mode), a request can result in one, several, or no hits. The results table obtained for each request shows data on percentages of GMO elements present in the listed GMO events (Figure 1, item G). Several action buttons or separations permit the user to have a best overview of the information displayed (Figure 1, items H, I, J, K).

#### Applications of the GMOseek matrix

The GMOseek matrix is helpful in GMO testing at different phases in the analysis. The numbers of GMOs and specific tests included in the matrix are flexible, and can be increased or decreased according to the available information, needs and specific situations. The frequency and distribution of the targets of screening modules among GMOs of the same and/or different species can be exploited in the design and subsequent use of the matrix.

The GMOseek matrix can assist in selecting the targets for a screening analysis, interpreting the screening results, checking the occurrence of a screening element in a group of GMOs or in the whole database, identifying gaps in the available GMO detection methods, or designing a decision tree. Some examples are presented below.

#### Data retrieval

Data on GMOs and GMO elements, including data on their frequency of occurrence are valuable information for GMO detection and analysis. Information for various types of purposes can be retrieved: search in the matrix (e.g. What GMO elements are present in the FG72 soybean?), comparison of information (e.g. What are the common GMO elements introduced in maize “Event 98140” and “Liberator L62” rapeseed?), or a list of the potential candidates present in a given sample (e.g. What are the potential candidate GMO events that have a signal with pFMV?). The functionalities provided together with the matrix help preparing GMO testing, explaining test results or improving the screening approach by choosing a more refined set of targets.

#### Selection of the targets for the GMO screening analysis

Guidelines for the preparation of GMO screening analysis using the matrix-based approach, according to the type and complexity of the samples in scrutiny, were recently released [10]. These guidelines are therefore highly recommended to consider before using the GMOseek matrix. In practice, the first step in GMO analysis is the use of a combination of screening methods to detect as many GMOs as possible. The p35S and tNOS screening targets are no longer sufficient to cover most commercialized GMO events [61], and GMO analysis requires a well-designed selection of qPCR screening targets for the matrix approach [10,12,62]. The GMOseek matrix allows a quick visual selection of the most frequent GMO elements marked by colour codes (see Figure 1, item A). The GMOseek matrix can fit the screening phase to the plant species in the sample under investigation, but does not propose cost-effective combinations as with the GMOtrack software [62]. Alternatively, a two-step screening approach can be performed before identification. In a first screening step, targets can be selected for those elements allowing a wide GMO coverage. The results of this first screening are then entered in the GMOseek matrix, and depending on the potential displayed candidates a second set of screening tests is selected as a function of its discriminating power. Results of this second screening round should decrease the number of required event-specific tests.

As an example of this functionality, combinations of genetic elements that could be used in screening phase are provided for the major food and feed crops subject to genetic engineering. The proposed combinations making use of frequently found genetic elements should be easily amenable in laboratories as most of these elements are targeted by already existing detection methods (PCR or qPCR). The proposed combinations cover a wide range (at least 80%, except for rice) of the GMO events known worldwide and 100% of the GMO events relevant to the EU regulations in food and feed [49,63] (Table 2).

**Table 2:** Most frequent genetic elements observed and proposed screening combinations in major food and feed crops

	**All GMOs from plant origin worldwide**			**GMOs from plant origin relevant to EU regulations**		
**Crop species**	**Most frequent genetic elements**	**Frequency**	**Proposed screening (coverage)**	**Most frequent genetic elements**	**Frequency**	**Proposed screening (coverage)**
**Rapeseed**	*tNOS*	39%	pTa29 (or pSsuAra or pSsuAra-bar)	*tNOS*	75%	
	*bar*	39%	p35S, *cp4*-*epsps*	*bar*	75%	tNOS , p35S, *CTP2*-*cp4epsps* (or tE9)
	*pSsuAra*	39%		*pSsuAra*/*pTa29*	75%	
	*pTa29*	39%				
	*pSsuAra*-*bar*	39%		*pSsuAra*-*bar*	75%	
	*p35S*	29%				
	*tg7*	29%		*tg7*	75%	
	*barstar*	29%		*barstar*	58%	
	*barnase*	29%	**(25/31 – 81%)**^**a**^	*CTP2*-*cp4epsps pTa29*-*barnase*	58%	**(12/12 – 100%)**
**Soybean**	*p35S*	38%	*p35S*, *tE9*, *cp4*-*epsps*, *tNOS*, *pat*,	*p35S*	44%	*p35S*, *pFMV*
	*tE9*	33%	*cryIAc*	*cp4epsps*	33%	
	*cp4*-*epsps*	33%		*CTP2*-*cp4epsps*	22%	
	*pFMV* (*pCmoVb*)	24%		*pat*	22%	
	*CTP2*-*cp4epsps*	24%		*tE9*	22%	
	*tNOS*	24%	**(18/21 – 86%)**^**b**^	*pFMV* (*pCmoVb*)	22%	**(6/9 – 66%)**^**g**^
**Maize**	*p35S*	80%	*p35S*, *tNOS*, *pat*	*p35S*	85%	p35S, tNOS
	*tNOS*	74%		*tNOS*	79%	
	*t35S*	60%		*pActin1*	52%	
	*pat*	56%		*pat*	39%	
	*35S*-*pat*	51%		*35S*-*pat*	39%	
	*pActin1*	42%	**(116/126 – 92%)**^**c**^	*cp4*-*epsps*	36%	**(33/33 – 100%)**
**Cotton**	*p35S*	57%	*p35S*, *pat*, *cry1Ac*, *nptII*, *m*-*epsps*	*p35S*	64%	*p35S*, *pat* (*or pUbiZM1*), *m*-*epsps*
	*tNOS*	50%		*tNOS*	55%	
	*cry1Ac*	43%		*cry1Ac*	55%	
	*nptII*	35%		*aad*	45%	
	*aad*	33%		*nptII*	45%	
	*t7S*	22%	**(39/46 – 85%)**^**d**^	*35S*-*nptII*	45%	**(11/11 – 100%)**
**Potato**	*tNOS*	88%	*tNOS*			EH92-527-1 is the only authorized potato
	*tE9*	38%				*event*: *no need for screening*
	*nptII*	69%				
	*cry3A*	69%				
	*p35S*	50%	**(13/15 – 87%)**^**e**^			
**Rice**	*p35S*	29%	*p35S*, *tNOS*, *cry1Ab*, *cry1Ac*, *CryIaB* /			*LL62 is the only rice that can be found in*
	*tNOS*	21%	*CryIac*, *pUbiZM1*, *m*-*epsps*, *tNOS*			*EU* (*LLP regulation*): *no need for screening*
	*cry1Ab*	19%				
	*cry1Ac*	17%				
	*cryIaB* / *cryIac*	17%				
	*pUbiZM1*	14%				
	*bar*	14%	**(27/42 – 64%)**^**f**^			

Most frequent genetic elements: The most frequent genetic elements in the major food and feed GM crop species worldwide (on the left) and for the GM events pertaining to the EU regulations (on the right). Frequency: The relative frequencies of each element respectively to all GMO of the same crop species, given as a percentage. Proposed screening (coverage): proposed combination of GM elements in order to achieve a good coverage (at least 50%) of all GM events in the crop species. The coverage is indicated by bold letters in brackets, as the ratio of detected GM events out of all currently known GM events.

Non-usable elements (short elements like leaders, plasmid t-DNA, elements originating from the same species) were not taken into consideration. Non-authorized and therefore non-covered events are marked by ^**a**^ event 73496; ^**b**^ 305423 and BPS‒CV127‒9; ^**c**^ 3751IR, 3243M, LY038, DAS-40278-9, HCEM485, 32138 and BVLA430101; ^**d**^ 1849, 19-51A, China cotton 1, China cotton 2, China cotton 3, GK12 and SGK321; ^**e**^ tBK50-13 and tBK50-60; ^**f**^ Kinuhikari 2, Zhuxian B, 86AB1, 86AS2, Bt-IR72, KA130, Kinuhikari 1, Nihonbare 16–2, Nihonbare 20–2, 21–3, Minghui 86a, Minghui 86b, PWC16, Tsuki-no-hikari H39 and H75. Some of the non-covered events can be detected by event specific assays validated by the European Union Reference Laboratory for GM Food and Feed (EU-RL GMFF, http://gmo-crl.jrc.ec.europa.eu/statusofdoss.htm).

#### Interpretation of the GMO screening results

The GMOseek matrix enables a simple and fast evaluation of test results after a screening and identification phase. After entering the detected pattern of GMO elements obtained during a first screening phase, all GMO events corresponding to that pattern can be selected using the sorting functionality in the “GMOseek” spreadsheet. Considering the composition of the product or the analytical request, the sorting functions can be restricted to different subsets (e.g. authorized/unauthorized). If the submission of a GMO element pattern does not generate any result, this indicates that no GMO in the GMOseek database shares the same pattern, possibly for one or a combination of the following reasons: (1) presence of several GMOs in the product, each containing only (a) part(s) of the GMO element pattern submitted in the query; (2) low level presence of some GMOs in the product (different limits of detection for the targets of the same GMO may result in unclear results); (3) an unlisted or unknown GMO in the database. In the first case, it is possible to obtain hits to a pattern by splitting this pattern in several complementary “sub-patterns”. This can be done by the activation of several rows in the GMOseek spreadsheet. The subdivision into sub-patterns can also be influenced by the strength of the signal. For example, a pattern with 2 ranges of Ct values for the screening targets tested could indicate the presence of several GMOs at different concentrations. The second case (low level presence of some events) may occur together with the first case, and the outcome of the screening analysis will be difficult to interpret. Highly varying amounts of ingredients in the sample and/or limits of detection of the qPCR assay should be considered. In the third case, where an unknown GMO can be present, it is highly recommended to perform further investigations, as described in Holst-Jensen *et al*. [10].

#### Selection of candidate GMO elements to develop new qPCR systems

The GMOseek matrix provides the most complete knowledge about the presence of screening elements in GMO events. However, qPCR assays do not exist for all the elements that could be targeted. For the development of a new screening assay, one has to consider the already existing screening systems and their coverage, the frequency of the GMO element present in GMO events, and the potential natural presence of the GMO element in plants in order to avoid donor organism detection. Increased coverage of GMOs and/or reduction of the number of identification tests can be achieved by a selection of screening elements for which methods could be developed.

The GMOseek matrix facilitates the choice of new singleplex or multiplex screening qPCR assays to develop. Besides information on the frequency of each GMO element, the GMOseek matrix distinguishes GMO elements and events (e.g. m-*epsps* and cp4-*epsps*, or elements in the Bt11 and Bt10 maize events). It links information on events, elements and plant species frequently found in food and feed, and enables a differential weighting of importance according to the authorization status. Therefore, the matrix helps the user to evaluate the usefulness of newly developed screening assays for a given GMO element. For instance, the growing number of Chinese rice events shows that the pUbi promoter and the *cry*1Ab gene are potential targets to detect such EU-unauthorized GMOs.

#### Precautions when using the GMOseek matrix

Using the matrix-based approach for GMO testing implies a careful check-up of the information contained into the matrix and also cautious interpretation of the results [9]. Detailed guidelines for the use of the matrix-based approach were recently provided and are not repeated in this work [10]. It is therefore strongly recommended to consider these recommendations before using the GMOseek matrix.

#### Experimental verification

Ideally, the information about the presence/absence of genetic elements in GMOs contained in the GMO matrix would be experimentally verified for each genetic element and GMO, indicating the reliability of the GMO matrix. Such verification is mainly dedicated to identify when a method targeting a given GMO element may not be able to detect a specific GMO event due to allelic variation in the targeted sequence, errors or incompleteness in the matrix. This implies that the reference material is from a reliable source and a detection method specific to the GMO element exists, what is possible only for a limited number of GMOs. Therefore, experimental verification tests were performed in priority for GMO elements in those GM plant species where their presence in some events was expected (and for which GM reference material of the considered plant species was available).

Experimental results mostly confirmed the data on the presence of GMO elements in GMO events (Table 1) showing that the approach taken for collecting data and quality control measures was appropriate. In a total of 530 tests (performed at least in duplicates) on 31 reference materials, 492 (93%) led to results in accordance with the presence of genetic elements in GMO events, as reported in the matrix.

In some cases, the results of the experimental verification may conflict with the theoretical information on the presence of a genetic element in a particular GMO. Unexpected results are most often linked to a suboptimal match of primer and probes due to unexpected differences in the DNA sequence between GMO events or in the design of the qPCR assay used to verify the inserted sequence. Looking in detail, GMO events can differ in the length, the nucleotide sequence or the order of the inserted genetic elements (e.g. a shorter introduced genetic element to which primers cannot hybridize).

An example is the p35S GMO element. Multiple assays targeting this element were published covering the whole promoter sequence. However, its exact sequence can differ between GMOs. Engineered plants can contain the full promoter sequence, duplicated copies of the promoter domain B or its enhancer sequence. Finally, some GMOs contain chimeric promoters made with the enhancer sequence of the p35S element, merged with a minimal promoter sequence from another origin. In the GMOseek matrix, all these elements were grouped under the term “p35S”. Not all the assays designed to detect p35S will be able to detect this large variety of target and it is therefore crucial to use an assay detecting the largest possible range of p35S elements (usually the enhancer sequence) or to know which elements are detected with a given assay. Two p35S specific assays used in this work target a region of the 35S enhancer present in most GMOs but absent in the maize 98140 event [50,51], hence the absence of signal observed when verifying the matrix (Table 1). By contrast, a third assay designed to target another region of the 35S enhancer [13] detects the element p35S in the event 98140 (Table 1).

The t35S terminator consensus sequence in most of the GMOs is generally 69 bp long and 78% A-T rich. These characteristics make it impossible to design specific qPCR primers and probes for this region. For verifying the presence of t35S in the matrix, an assay targeting a larger region (118 bp) was used. The obligation to use a less specific assay explains the late signals obtained for the DAS19122 maize, CBH351 maize, and T45 rapeseed events, and the bad amplification curves obtained for the Bt176 maize event (Table 1). In maize event 3272, the t35S element was not detected using this t35S qPCR assay although this event supposedly contains a 70 bp t35S segment. Considering the small size and high A-T content of this segment, a t35S specific test may be inefficient for event 3272. A total of five tests led to misinterpretation of the absence/presence of t35S. Together, these two examples stress the need for verification/confirmation by experimental analysis when creating a reliable GMO screening matrix.

When comparing the theoretical data with experimental results, precaution is advised concerning the characterization of the reference material used. Even CRMs are only characterized for the presence of certain GMO, but not for the absence of others. The presence of non-declared GMO in CRMs has been already observed [10,13]. Also in our study, traces of non-declared GMO material than the certified GMO event were sometimes observed, resulting in unexpected signals (see Table 1, “source of impurity”). While verifying the matrix, 28 tests results (5% of total results) initially not complying with the information in the matrix could be explained by GMO impurities identified in the tested reference material. Three additional false-positive results with very late signal (high Cq values) were observed for two reference materials. However, the large difference in Cq values between the expected signals and these false positive signals are suggesting the presence of GMO impurities that were not identified. Together, 38 out of the 530 experimental results (7%) differed from the matrix data. From these, all the 31 false-positive test results could be explained by the presence of GMO impurities, identified in most cases. Four ambiguous results (late positive or bad signals) were obtained with t35S. One false negative result was obtained with the t35S specific method due to the small t35S sequence present in the tested material. Finally, two other false negative results were explained by the absence of the targeted sequence in p35S. All deviations from the matrix data could therefore be explained.

#### Interpretation of results

The explanations above stress the need for the verification/confirmation by experimental analysis when creating a reliable GMO screening matrix. It is strongly recommended to carefully characterize the reference materials with event-specific assays prior to use them for matrix experimental verification. Each user should therefore experimentally verify its set of GMO element-specific assays and correct the GMOseek matrix accordingly. In that sense, a large collaborative study rather than experimental verification by individuals would be of real help to harmonize the use of the matrix-based approach and certify the quality of GMO detection following this strategy.

Other precautions regarding the use of the matrix-approach for GMO analysis were previously described [10,13]. They mainly deal with the practical and absolute limits of detection of the assays used in a given sample, the use of screening elements originating from the donor organism of a GM element, the need to precisely identify the different crop species present in a complex sample before interpreting the results of the GMO screening phase using the GMOseek matrix.

## Conclusions

The GMOseek matrix currently constitutes the most complete and comprehensive database gathering information on 328 GMO events with 247 different elements present in their respective GM constructs. Handling all these data requires a user-friendly system. For this reason, the herein described Excel environment offers several functionalities facilitating data searches, the development of GMO detection strategies, data analysis, and decision making for GMO detection. The GMOseek matrix also provides information useful for the selection of additional screening targets for which new singleplex or multiplex qPCR detection assays need to be developed. It serves as a comprehensive tool useful to GMO testing laboratories and control authorities. The flexibility of the GMOseek matrix facilitates its adaption for use in many countries, taking into account their respective legislations. The authors strongly recommend that users of the GMOseek matrix consider the recommendations and limitations of the matrix approach [10].

In the future, the data of the GMOseek matrix will be integrated into the larger web-based EUginius database [64,65]. EUginius (European GMO Initiative for a Unified Database System) will be a user-friendly retrieval system providing information on GMOs concerning their phenotypic and molecular characterisation (DNA sequences), detection methods, reference material, as well as their GMO world-wide regulatory status. The GMOseek matrix can be used also with a so-called GMOseek algorithm, another central part of the GMOseek project (http://kt.ijs.si/software/GMOtrack/GMOseek.html). This mathematical algorithm selects from the matrix an optimal set of genetic elements that need to be targeted for the detection of GMOs and should allow streamlining different GMO screening applications.

Building such a matrix was a time consuming task and would not have been possible without the international collaboration of the GMOseek partners. Further effort will be needed to maintain and update the matrix with new GMO events and genetic elements, and to experimentally confirm the presence of genetic elements in GMOs. It will require developing new detection methods and, when certified GMO reference material is not available, using precisely defined alternative reference material. In order to avoid duplication of work and to ensure harmonization of GMO testing, the authors are calling for a broader collaboration of the laboratories involved in GMO detection within and outside of Europe.

## Availability and requirements

For its full functionality, the GMOseek matrix requires an Excel version 2007. Earlier Excel versions will show only a part of the information. The GMOseek matrix database is freely accessible on the internet, including a “User’s manual” with detailed instructions from http://www.cra.wallonie.be/en/19/the-projects/296.

## Competing interests

All authors are professionals employed by the Bavarian Health and Food Safety Authority (LGL, Germany), Walloon Agricultural Research Centre (CRA-W, Belgium), Federal Office of Consumer Protection and Food Safety (BVL, Germany), Institute for Agricultural and Fisheries Research (EV ILVO, Belgium), European commission, Directorate General Joint Research Centre, Institute for Health and Consumer Protection (EC JRC-IHCP, Italy), Scientific Institute of Public Health (WIV-ISP, Belgium), National Institute of Biology (NIB, Slovenia), and as such do not have any interests that may conflict with the contents of the present article.

## Authors’ contributions

Data collection, verification and maintenance: LGL, CRA-W, NIB, EV ILVO, WIV-ISP, EC JRC-IHCP. Experimental verification: CRA-W and NIB. Elaboration of the Excel-based tools: CRA-W. All authors have read, commented and approved the final manuscript.

## Supplementary Material

Additional file 1 **GMOseek_Matrix-Program_version_13-1.xls, Excel program version 2007, description of the file functionalities see Additional file**2**, XLS;**http://www.cra.wallonie.be/en/19/the-projects/296. Click here for file

Additional file 2 **User Manual GMOseek Matrix.pdf, user manual for the GMOseek Matrix program, additional documentation, PDF; **http://www.cra.wallonie.be/en/19/the-projects/296. Click here for file
