# Supplementary material for: The GMOseek matrix: a decision support tool for optimizing the detection of genetically modified plants
Source: BMC Bioinformatics. 2013 Aug 22;14:256. doi: 10.1186/1471-2105-14-256 (PMC3765097; doi:10.1186/1471-2105-14-256)
Supplement: Additional file 2 — User Manual GMOseek Matrix.pdf, user manual for the GMOseek Matrix program, additional documentation, PDF; http://www.cra.wallonie.be/en/19/the-projects/296. [file 1471-2105-14-256-S2.doc]

**GMOseek Matrix user manual**

**Before starting...**

**Open the GMOseek Matrix**

The GMOseek matrix requires a version of Excel 2007 or higher in order to avoid compatibility problems. Indeed, some older versions of the Excel program can not deal with the number of columns that are required. Before using the GMOseek matrix, it **first has to be saved on your computer**. Secondly, you have to **activate** the authorisation to use the **macros** present in the GMOseek matrix (click on “Enable content” in the Excel versions of 2007 and 2010). Without these two actions, the program will not work.

**The GMOseek matrix**

1. **The different sheets of the Excel file**

This application is a complex tabulated data matrix compiled in a Microsoft Office Excel format. The so-called “GMOseek matrix” facilitates a quick and easy overview of all genetic elements (hereafter named “GMP elements”) used and provides information on their presence or absence in a set of GMP events. Raw data are collected and presented in the sheet named “**Matrix**”. The “**Matrix**” sheet provides an allocation of all listed GM elements and their presence or absence in the tabulated GM events.

To handle this complex data collection, an user-friendly system for data retrieving is offered with several functionalities facilitating informative search, comparison of data and decision-making tools in the sheet “**GMOseek**” (figure 1). The retrieving system extracts data collected in the “**Matrix**” and the “**Listed Events**” spread sheets through different functionalities. The sheet “**Listed Events”** is an alphabetical classification of GM events, Unique Identifier and plant species names that are used for the display of the names in the different drop-down menus of the GMOseek sheet .

The sheets “**Legal use”,** “**Authors”** and **“Info - Tools”** supply general information on how to cite this database and the Excel application for reports or publications, on who are the persons having participated to the preparation of the matrix, and on how to download the user manual of the GMOseek Matrix.


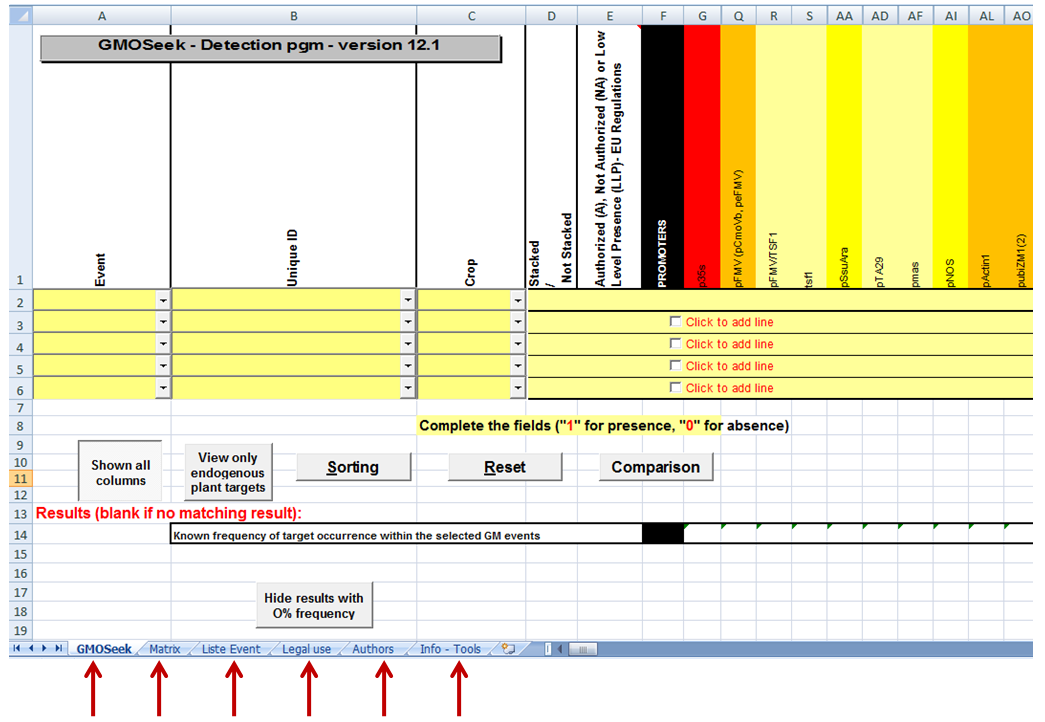
**Figure 1: Overview of the GMOseek Matrix**

- 1. **Colour code used in the GMOseek Matrix**

In the working sheets ‘**GMOseek**’ and ‘**Matrix**’ the frequency of each GM element relative to all listed events is indicated by the colour code of the headers of the columns. This helps the user weighing the importance of a GM element. Four colours (figure 2) are used to indicate to which frequency class the element belongs: red colour for an occurrence greater or equal to 30%, orange for an occurrence between 29% and 10%, yellow for an occurrence between 9% and 5%, pale yellow for an occurrence between 4% and 2%. Uncoloured cells contain elements with an occurrence of less than 2%.


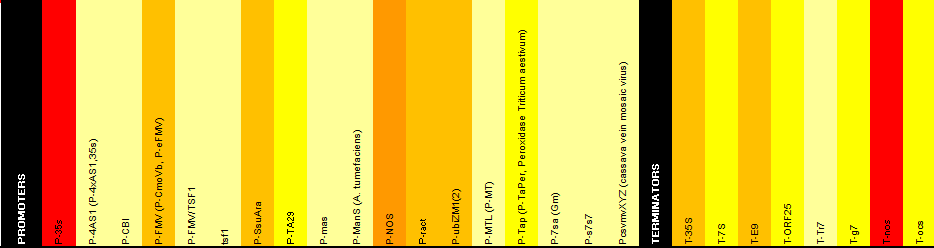


Figure 2: Overview of the colour code of some elements

Black columns separate the various types of GMP elements like terminators, promoters, open-reading frame segments, miscellaneous elements, junctions and plants (figure 2).

- 1. **Endogenous targets**


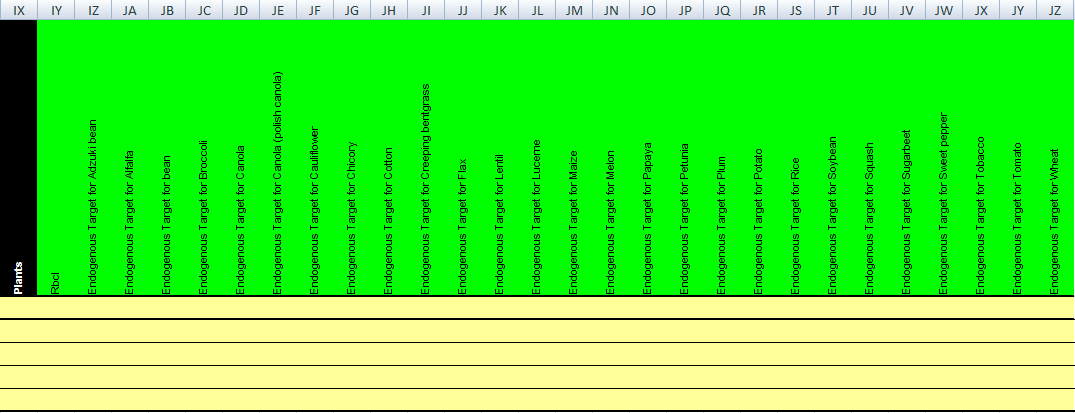
At the end of the screening element columns, a list of endogenous plant targets is added. As several targets can be used for a same plant species (e.g. invertase, adh, hmg for maize), targets are listed under the name “endogenous target” (e.g. endogenous target for maize). Endogenous plant targets headers are coloured in green (figure 3).

Figure 3: Overview of the section dedicated to the endogenous plant targets

Clicking on the button “**view only endogenous plants targets**” (figure 1) gives a rapid access to these plant endogenous target columns. This can be useful if you want to link the results of your PCR tests to a defined set of plants species. By pressing the inverse **“Return”** button the whole view is restored.

- 1. **Display a defined selection of GM elements**

**The GMOseek Matrix contains a large number of columns. In many applications of the matrix, low frequency GM elements are less interesting because of their low coverage in detecting GMP. For this reason,** elements with a relative frequency lower than 2% can be masked with the functionality button “**Hide low frequency columns**” to allow a more readable table. A detailed view on all GM elements is restored by pressing the button “**Show all columns**”.

For customized applications, the user can redefine which part of the GM elements (columns) to be shown or hidden. For this option, the following operations have to be performed:

- In the Excel toolbar, click on “Formulas”
- Select “Name Manager”. The screen presented in figure 4 will appear.
- Select the “hidden” Name of the “Workbook” scope


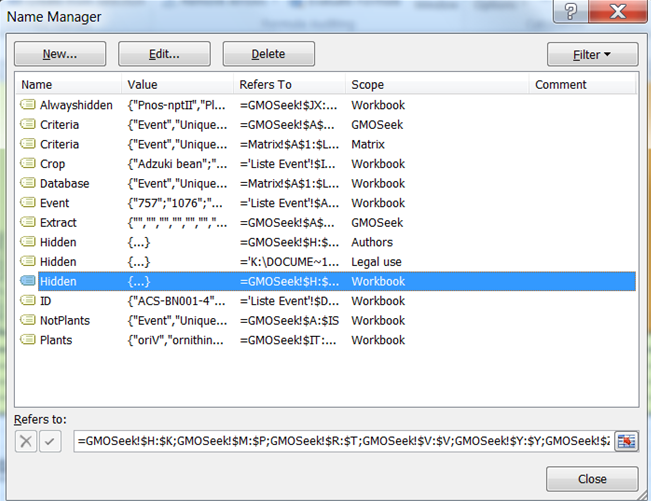


Figure 4: Content of the Name Manager.

- Erase the information in the cell “Refers to” and realize a new selection of the columns to hide
- Close the Name manager and save the modified version of the Excel file.
  1. **Information in the cells of the Matrix**

The presence of a given GMP element or an endogenous target for a given event is marked by “**1**”, the absence is marked by “**0**”. Unfilled cells are not verified yet. For these empty cells, the information on the presence or absence of a GM element is either not available or the correctness of the information could not be checked. Unfilled cells will be completed in updated versions of the matrix once reliable information confirming the presence or absence will be available.

**Warning:** Please be aware, that unfilled cells are not taken into account when sorting parameters are based on presence (1) or absence (0).

1. **How to use the GMOseek Matrix**
   1. **Search for information in the GMOseek Matrix**

If several consecutive queries are to be made, use the “**Reset**” button to erase the data of a previous demand before a new search is done.

A search can be done on the basis of a name (event name, unique identifier or plant name), a status (Authorized, Not Authorised, Low level presence) or in function of the GM element(s) present.

The second row of the **‘GMOseek’** sheet (highlighted in yellow) is an interrogation row and is used for introduction of the selective parameters. Several interrogation rows can be activated. After selection of the appropriate elements, a left-click on the **‘Sorting’** button will retrieve all the GMP events of the database corresponding to the exact request.

- - 1. ***Search for information about GMP events by the drop-down menu***

By left-clicking once in the field “**Event”**, “**Unique ID**”, “**Crop”**, “**Stacked / Not Stacked**” or “**Authorized /Not Authorized/ Low Level Presence**” (relative to EU regulation) in the yellow highlighted row, a drop-down menu appears in these cells. A second left-click on this “drop-down menu” provides the list of selectable elements classified in alphabetical order (see figure 5).


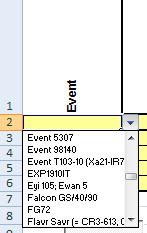

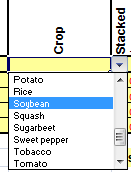

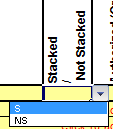

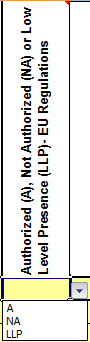


Figure 5 : Examples of information in the drop-down menus of “Event”, “Crop”, “Stacked/Not Stacked” and status in EU

For the cells in which unique ID, event or crop names, can be introduced, you can start typing the name and the program will help the user by proposing a name that is adapted in function of  the already introduced sequence of letters, if it does not correspond to what is expected continue to type additional letters to the sequence until the correct name is reached.

*The case of stacked events*

The application allows to find out all stacked events containing a particular event (e.g. all the stacked events containing the MIR604 cassette). Therefore, put a star in front of the name of the GM event (e.g. *MIR604). By a click on the sorting button, the program will list all the stacked events in which MIR604 is involved.

- - 1. ***Search for GMP events by the GMP elements***

For the retrieval of a subset of GMP events according to a defined presence/absence pattern of elements, this pattern has to be encoded by “1” (presence) or “0” (absence) in the cells of the interrogation’s rows, under the element columns’ headings.

Adding a “1” in a cell implies a mandatory presence of that GMP element in the selected GMP events; reversely adding a “0” will exclude GMP events having that GMP element; empty cells are not taken into account for the sorting option. Results appear after a click on the sorting button.

A combination of presence or absence of GMP elements in a same line has to be understood as a “AND” function (e.g all the GMP events having both p35S and tNOS).

For retrieval of the GMP events having at least one of the selected GMP elements (e.g the GMP events having p35S or tNOS), the cell “**Click to add line**” has to be activated, and the appropriate alternative parameters introduced on this new line (figure 6). Once activated, the text “**Click to add line**” disappears in the new line. Parameters introduced on separate lines are understood as an “OR” function. Up to four additional request lines can be activated.

The activation of “**Click to add line**” can also be used for an interrogation concerning the status of the GM events (Authorized, Not Authorized and Low Level Presence) in order to select several statuses.


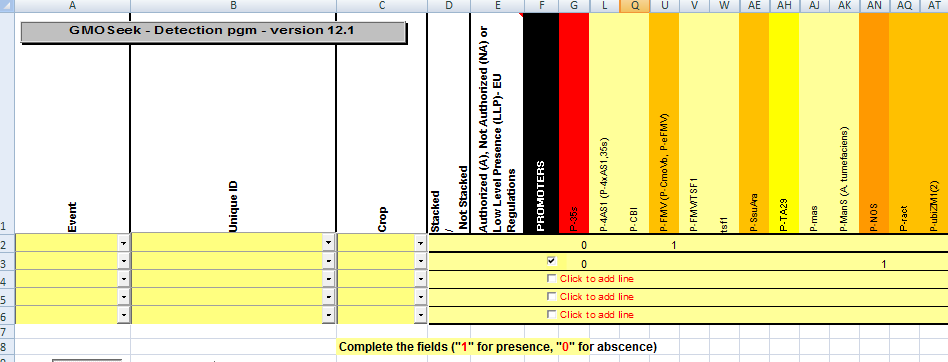
Figure 6 : Introduction of parameters in the GMOseek Matrix and activation of a second line of parameters for selection of potential candidates.

- 1. **Relative frequency of GM elements in a defined selection of GMP events**

A request (see 2.1.1) can result in one, several or no hit. The upper row of the result table named at the left side as “Known frequency of target occurrence within the selected GM events” highlights the percentage of sorted GMP events having the considered GMP element (figure 7). This specific percentage value reflects the relative occurrence of a GM element in all GM events of the selection.

**Warning:** Mind that the “known frequency of occurrence within the selected GM events” takes into account only the cells filled with “1”, the total number of cells containing either “1”, “0” or no information. This frequency could be underestimated if a cell with no information (considered as negative in the frequency calculation) actually contains the specific element.

This information is useful 1) to choose new elements to focus on in a second screening phase of the routine analysis and 2) to restrict the number of event-specific tests (during the identification phase), or 3) to define new screening methods to be developed for wider coverage of the GMP events.

In the generated selection the button “**Hide results with 0% frequency**” offers the option to hide all GM elements with an occurrence of 0 %,and therefore to provide a better overview of all informative GM elements.

**Note**: However, some columns indicating 0% will not disappear due to the fact that the real value is not 0% but is below 0.5%. The real frequency can be obtained by adding a decimal
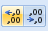
. This option can be reversed by activating the button “**Show all results**”. For a better overview, the functions “”**Hide results with 0% frequency**” and “**Show all columns/ Hide low frequency columns**” can even be combined.


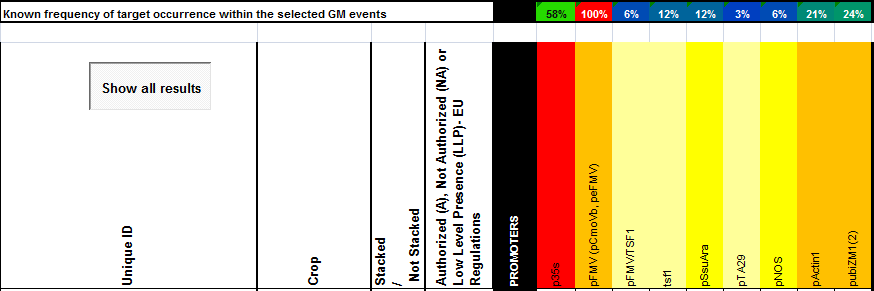


Figure 7 : “Target frequency within the selected events” for pFMV presence.

In this query, pFMV has a 100% of occurrence, while 58% of the sorted GM events have also a p35S and 6% have a pNOS.

If the user wants to have information on the relative occurrence of all GM elements in the whole database, sorting information without defining selective parameter (just a click on the sorting button) has to be performed.

- 1. **Comparison of information**


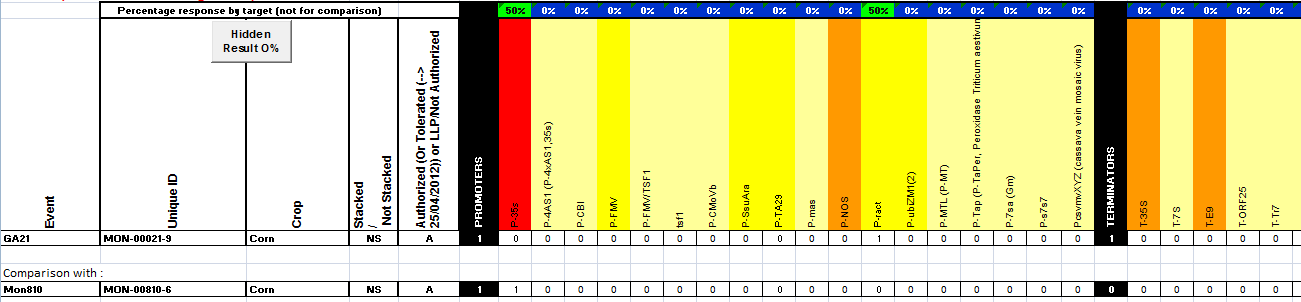
The **‘Comparison’** button allows the comparison of one GMP event with another one (or eventually with the results of a search). After selection of the first GMP event either in the ‘Event’ or ‘Unique ID’ drop-down menus, press the ‘Sorting’ button. Then a second selection can be done. This second selection is not limited to an entry of the GMOseek database but can be extended to a combination of defined parameters which might for instance result from a screening assay. This allows the operator to check visually the similarities or differences between the patterns.

Figure 8 : Overview of the display of a comparison between two GM events

- 1. **Application strategies**

The GMOseek matrix is helpful for GMP detection through several strategies, at different phases of the analysis. It can be used to select the targets for a screening analysis, to interpret screening results and to select interesting elements in function of their occurrence in the database. Applications are described in the publication:

# Block A. and Debode F., Grohmann L., Hulin J., Taverniers I., Kluga L.,Barbau-Piednoir E., Broeders S., Huber I., Van den Bulcke M., Heinze P., Berben G., Busch U., Roosens N., Janssen E., Žel J., Gruden K., Morisset D. (2013) The GMOseek matrix: a decision support tool for optimizing the detection of genetically modified plants. *BMC Bioinformatics*

- 1. **Suggestions**

Any suggestions or information to complete the matrix is welcome. In case of information permitting to fill a gap, please send the complete source of information (peer-reviewed paper, official document, description of application,...) in order to permit a check before entering the data. Contact : Frederic Debode, CRA-W, Belgium (f.debode@cra.wallonie.be).
